# Sunlight-Driven Emission of Eu^3+^ and Tb^3+^ Nalidixate Complexes: Achieving Visible Red and Green Light in PMMA Films

**DOI:** 10.1021/acsomega.6c04688

**Published:** 2026-06-30

**Authors:** Vitor Waldes, Paulo R. S. Santos, Gabriel A. M. de Oliveira, Israel P. Assunção, João Honorato de Araujo-Neto, Iran F. Silva, Pedro Miranda, Maria Cláudia F. C. Felinto, Carlos Lodeiro, Oscar L. Malta, Hermi F. Brito

**Affiliations:** † Department of Fundamental Chemistry, Institute of Chemistry, University of São Paulo, 05508-900 São Paulo-SP, Brazil; ‡ Education, Science and Technology Federal Institute of São Paulo, 01109-010 São Paulo, Brazil; § Department of Chemistry, University Federal of Paraíba, 58051-970 João Pessoa-PB, Brazil; ∥ Nuclear and Energy Research Institute−IPEN/CNEN, 05508-000 São Paulo-SP, Brazil; ⊥ BIOSCOPE Research Group, LAQV-REQUIMTE, Chemistry Department, NOVA School of Science and Technology (FCT NOVA), Universidade NOVA de Lisboa, Caparica 2829-516, Portugal; # PROTEOMASS Scientific Society, 2825-466 Costa de Caparica, Portugal; ∇ Department of Fundamental Chemistry, 28116 Federal University of Pernambuco, 50670-901 Recife-PE, Brazil

## Abstract

In this work, a series of complexes with the general formula [RE_2_(nal)_6_(H_2_O)_3_]·9H_2_O (RE^3+^: Eu, Gd, and Tb) was successfully synthesized. The crystal structure of the Tb^3+^ compound was determined by single-crystal X-ray diffraction, revealing a bimetallic arrangement with distinct coordination environments. Photophysical properties indicate that the Tb^3+^ complex shows efficient sensitization through the S_1_ level of the *nal* ligand. The presence of an LMCT band in the Eu^3+^ complex does not result in significant luminescence quenching. This can be attributed to hydrogen-bonding interactions within its structure and to the *nal* ligand’s high capacity as an indirect luminescence sensitizer. As a result, the Eu^3+^ complex exhibits a relatively high intrinsic quantum yield value (∼24%) for a highly hydrated system. The PMMA:(1%)­Eu^3+^ film shows a higher yield of highly luminescent films (54%) than the complex. In addition, the PMMA doped with Eu^3+^ and Tb^3+^ ions exhibits red and green emission under solar exposure. To the best of our knowledge, this behavior has not yet been reported in the scientific literature, considering the same ligand. These findings highlight the potential of these photonic materials as candidates for luminescent solar concentrator (LSC) devices.

## Graphical abstract

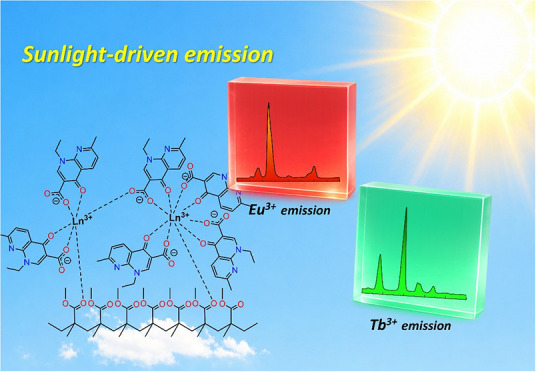

## 1 Introduction

The search for alternative energy sources has grown in recent decades due to the possible depletion of fossil fuels, the main energy source used by humanity.^1^ Thus, the use of solar energy through the development of solar panels represents a solution, as it is infinite and fully renewable. However, their use is limited, as they have a low conversion efficiency of sunlight into energy, around 35%.^2,3^ This problem can be overcome by using enhanced luminescent materials, such as luminescent solar concentrators (LSCs). These devices are composed of a waveguide polymer that converts high-energy photons into lower-energy photons.^1^ The process increases the durability and efficiency of photovoltaic cells positioned at the edges of the device.

In this way, coordination compounds based on trivalent rare earth ions (RE^3+^) have been intensively researched due to their multiple applications, such as optical markers,^4,5^ temperature sensors,^6,7^ diagnostic medicine,^8,9^ anticounterfeiting tags,^10,11^ and as LSCs.^12,13^ This variety of applications is due to their intrinsic electronic structure, in which the 4f electrons are shielded from the chemical environment by the filled 5s^2^ and 5p^6^ subshells, resulting in narrow emission and absorption bands of atomic character, leading to a higher color purity and easier spectral data interpretation.^12,14^ The 4f-4f transitions are forbidden by the Laporte rule, thus having a long lifetime and low molar absorptivity coefficients.^15^ One of the ways to overcome such drawbacks is the coordination of the RE^3+^ ions with some chromophore organic ligands, which can act as indirect luminescence sensitizers, absorbing the energy and transferring it efficiently to the metal ion, a phenomenon known as *antenna effect*,^16^ first reported by Weissman in 1942.^17^

The nalidixate organic ligand (Figure 1a), a derivative of nalidixic acid, was adopted as a luminescence sensitizer. Such compound is the main component of a synthetic medication group known as quinolones, a class of pharmaceuticals exhibiting several biological activities, being antimicrobial, antiviral, antiparasitic, anti-inflammatory, anticonvulsant, anticancer, and neuroprotective, in addition to having diuretic activity, and are widely used to treat genitourinary infections, prostatitis, respiratory diseases, as well as skin and soft tissue infections and neuropathic pain.^18,19^

**Figure 1:**
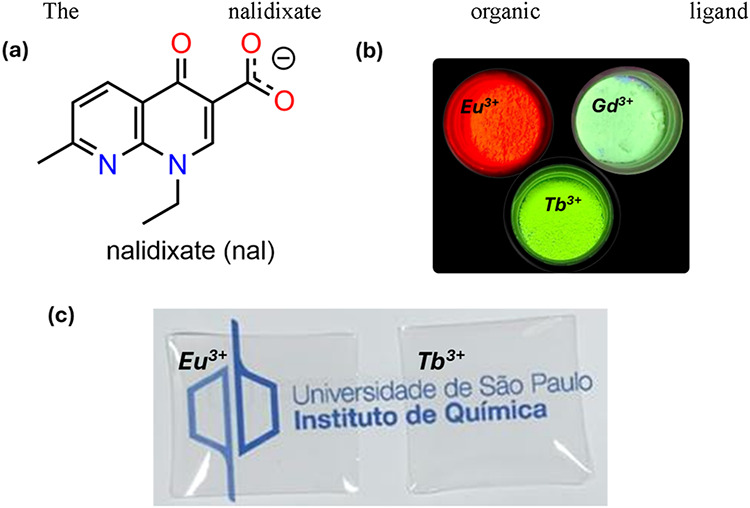
(a) The molecular structure of nalidixate ligand (nal). (b) Photograph of the complexes powder, upon artificial UV light. (c). Photograph of the PMMA 1% (w/w) films, upon artificial white light.

Besides, polymeric organic systems exhibit versatility, thanks to their physical and chemical characteristics, such as mechanical resistance, low production cost, and flexibility,^12,20^ leading them to a wide range of applications, such as in the security area,^21,22^ agriculture,^23,24^ medicine^25,26^ and solar energy^12,16^ and numerous other applications. For example, the poly­(methyl methacrylate) (PMMA) polymer exhibits interesting properties that make it a strong candidate for synergistic use with coordination compounds based on rare earth ions, such as UV light resistance, reasonable resistance to chemicals, and also being promising for photonic applications in optical devices, pneumatic actuators, sensors, analytical separation, and conductors.^27^

The literature reports luminescent polymeric systems under solar irradiation based on Ln^3+^ ions, such as the Eu^3+^-based β-diketonate complexes, which exhibit high emission intensity upon exposure to sunlight.^28^ Similarly, systems containing Tb^3+^ ions coordinated to a carboxylate ligand (such as the flufenamate anion) have also been described when doped into polymeric matrices that exhibit luminescence under solar irradiation.^12,20^ Nevertheless, the present work distinguishes itself by proposing systems capable of exhibiting emission under solar irradiation for both Eu^3+^ and Tb^3+^ ions with the same organic ligand, a feature that, to the best of our knowledge, remains unexplored in the literature.

Thus, this work aims to synthesize, characterize, and investigate the photophysical properties of the [RE_2_(nal)_6_(H_2_O)_3_]·9H_2_O complexes, incorporating RE (Eu^3+^, Gd^3+^, and Tb^3+^) ions as metal centers, in a PMMA matrix. The Eu^3+^- and Tb^3+^-based ones exhibit red and green intense luminescence of powder samples (Figure 1b), despite the considerable number of H_2_O molecules in their structures, which act as high-energy oscillators. Also, the transparent PMMA:(1%)­[RE_2_(nal)_6_] films (RE^3+^: Eu^3+^ and Tb^3+^) (Figure 1c) are highly luminescent when excited under UV light and under sunlight exposure for both RE^3+^ complexes by using the same *nal*-ligand, which, to the best of our knowledge, is unprecedented in the literature. These polymer films can be applied as light converters, including luminescent markers, optical sensors, and solar luminescence concentrators (LSCs).

## 2 Experimental Procedures

### 2.1 Synthesis of the [RE_**2**_(nal)_**6**_(H_**2**_O)_**3**_]·9H_**2**_O Complexes

The reagent nalidixic acid (Hnal, 99%), 1-ethyl-7-methyl-4-oxo-1,4-dihydro-1,8-naphthyridine-3-carboxylic acid, was purchased from Sigma-Aldrich and used without further purification. The Ln_2_O_3_ lanthanide oxides Eu_2_O_3_ and Gd_2_O_3_ were purchased from CSTARM, all with 99.99% purity, while Tb_4_O_7_ was purchased from Rhodia, also with 99.99% purity. The oxides were converted into lanthanide nitrates (where RE: Eu^3+^, Gd^3+^, and Tb^3+^), through the reaction reported in the literature^20^ of the oxides Eu_2_O_3_, Gd_2_O_3_, and Tb_4_O_7_ with nitric acid (65% w/w) (*d* = 1.39 g·cm^–3^) until reaching a pH close to 6, whereas for the terbium oxide, the use of hydrogen peroxide (50% w/w) (*d* = 1.2 g·cm^–3^) was necessary for the complete reduction of tetravalent terbium ions to trivalent ones. The solutions were stirred and heated to 60 °C. After this step, they were filtered and placed in a porcelain capsule for evaporation in a water bath until complete solvent evaporation, yielding the solids RE­(NO_3_)_3_·5H_2_O.

The preparation of the [RE_2_(nal)_6_(H_2_O)_3_]·9H_2_O complexes was carried out by mixing in a one-pot method the nitrates obtained in the previous step with nalidixic acid and an aqueous sodium hydroxide solution. This step began with the deprotonation of 3 mmol of nalidixic acid in an aqueous medium by dropwise addition of a sodium hydroxide solution (1.0 mol·L^–1^), while heating to 80 °C until the solution reached a pH close to 8. Subsequently, the RE­(NO_3_)_3_·5H_2_O precursor, once dissolved in distilled water, was added to the solution at a 1:3 metal-to-ligand ratio, immediately forming a solid, which was then heated to 80 °C under stirring for 2 h. Afterward, the mixture was filtered, and the complexes retained on the filter were heated to 50 °C in the oven until complete solvent evaporation, yielding solid samples that were soluble in methanol and ethanol at room temperature, but only in isopropyl alcohol at 70 °C. The synthesis of these compounds had yields of 85, 87, and 86% for the complexes based on Eu, Gd, and Tb, respectively.

### 2.2 Preparation of the Doped Polymeric Film Systems

The PMMA:(1%)­[RE_2_(nal)_6_] films (RE^3+^: Eu^3+^, Gd^3+^, and Tb^3+^) were prepared containing 1% by weight of the complex, following the methodology reported by Canisares *et al*.^29^ Approximately 5 mg of the complex were previously dissolved in MeOH and added to a chloroform solution containing 500 mg of PMMA, and, after about 5 mL of the solvent remained, the mixture was deposited on a glass substrate and covered with a Petri dish in a chloroform vapor-saturated atmosphere.

The analyses of carbon, hydrogen, and nitrogen (CHN) content were performed at the Elemental Analysis Laboratory of the Analytical Center of the Institute of Chemistry—University of São Paulo (USP), using a PerkinElmer CHN-2400 instrument. Thermogravimetric analyses (TG) were performed from 25 to 900 °C under a dynamic synthetic air atmosphere, with a flow rate of 50 cm^3^·min^–1^ and a heating rate of 10 °C·min^–1^, on a Shimadzu TGA-50 thermobalance. Fourier transform infrared spectra (FTIR) were obtained on a Bruker VERTEX 70v spectrometer using a Thallium Bromoiodide (KRS-5) cell in the transmission mode. The powder X-ray diffraction was obtained with a Miniflex Rigaku diffractometer, using Cu Kα_1_ radiation (30 kV and 15 mA) in 2θ, with a pass time of 0.05 s and a range of 3–50°.

Diffuse reflectance spectra (DRS) were recorded from 300 to 800 nm with a Shimadzu UV-2600 equipment containing an integrating sphere, using BaSO_4_ as a standard. The acquisition of photoluminescent excitation and emission spectra was performed through luminescence spectroscopy analysis, being recorded at room temperature (∼300 K) and under liquid nitrogen (∼77 K), using a Horiba Jobin Yvon Fluorolog-3 spectrofluorometer with a single Spex 1680 monochromator, a photomultiplier, and a 450 W xenon lamp as the excitation source. The fluorometer light intensity was controlled by selecting an excitation and emission slit aperture from 0.5 to 2.0 mm and a detection angle of 22.5° (front face). In contrast, the emission decay curves were obtained at ∼300 and ∼77 K using a SPEX 1934D phosphorimeter, which is coupled to the spectrofluorometer. The emission spectra of the PMMA:(1%)­RE^3+^ films were obtained using an Ocean Optics fiber-optic equipment, with a 1 mm diameter, connected to an Ocean Optics QE65000 spectrometer with a resolution of up to 1 nm by exposing the doped films to the direct Sunlight irradiation in an open external environment.

Single crystals suitable for X-ray diffraction were mounted on a loop using Paratone oil. Data collection was carried out on a RIGAKU Synergy-S diffractometer equipped with a microfocus Cu Kα radiation source. The structure was solved by intrinsic phasing using SHELXT^30^ and refined by full-matrix least-squares on F^2^ using SHELXL,^31^ as implemented in Olex2.^32^ All non-hydrogen atoms were refined anisotropically, and hydrogen atoms were placed in calculated positions and refined using a riding model. Disordered regions were modeled using split positions with refined occupancies and restrained using appropriate geometric constraints (e.g., DFIX). A significant amount of solvent was found to be highly disordered and could not be satisfactorily modeled. Therefore, its contribution was treated using a solvent-masking procedure and was excluded from the final refinement. Molecular graphics and visualization were performed using Mercury software.^33^

## 3 Characterization of the RE^3+^ Systems

The experimental and calculated data for the elemental analysis (CHN) of the complexes showed good agreement with [RE_2_(nal)_6_(H_2_O)_3_]·9H_2_O general formula, as follows for calc. - for [Eu_2_(nal)_6_(H_2_O)_3_]·9H_2_O; C_72_H_90_Eu_2_N_12_O_30_: C: 45.34%, H: 4.76% and N: 8.81%; found −C: 46.13%, H: 4.37% and N: 8.67%; [Gd_2_(nal)_6_(H_2_O)_3_]·9H_2_O; calc. for C_72_H_90_Gd_2_N_12_O_30_: C: 45.09%, H: 4.73% and N: 8.76%; found - C: 44.77%, H: 4.55% and N: 8.58%; [Tb_2_(nal)_6_(H_2_O)_3_]·9H_2_O; calc. for C_72_H_90_Tb_2_N_12_O_30_: C: 45.01%, H: 4.72% and N: 8.75%; found - C: 46.1%, H: 4.41% and N: 8.87%.

The thermogravimetric study was conducted using 10 mg samples, namely the [RE_2_(nal)_6_(H_2_O)_3_].9H_2_O complexes (RE: Eu^3+^, Gd^3+^, and Tb^3+^) and the free *Hnal* ligand, in a temperature range from 25 to 900 °C, under a dynamic synthetic air atmosphere at a flow rate of 50 cm^3^·min^–1^, using a heating rate of 10 °C·min^–1^. The thermogravimetric curves of the complexes are illustrated in Figure S1a, and show very similar behavior due to the similarity of the ionic radii of the three Eu^3+^, Gd^3+^, and Tb^3+^ ions studied. The analysis of the *Hnal* curve shows a single major thermal decomposition event that starts at around 195 °C and reaches a peak at approximately 300 °C. The TG curves of the [RE_2_(nal)_6_(H_2_O)_3_]·9H_2_O complexes exhibit very similar behavior (Figure S1a), with all of them showing a mass loss of about 11% of their total mass due to water release. In this case, it is not possible to distinguish which water molecules are coordinated and which are crystallization water, totaling 12 water molecules in the structure of the three compounds.

For all three complexes, the thermal decomposition of the organic moiety occurs at approximately 260 °C and is completed at around 550 °C, corresponding to about 70% of the total mass loss. The remaining residue consists of rare-earth oxides (Eu_2_O_3_, Gd_2_O_3_, and Tb_4_O_7_), accounting for approximately 21% of the total mass. Additionally, mass loss is observed in the three complexes at room temperature (∼25%), a phenomenon explained by the lower stability of the channel water molecules, an issue that will be further addressed below, together with single-crystal X-ray diffraction (SC-XRD).

Analogously, TG curves of the PMMA:(1%)­[RE_2_(nal)_6_] (RE: Eu^3+^ and Tb^3+^) films, compared with that of the undoped PMMA film (Figure S1b), are relatively less thermally stable, possibly due to the instability of the polymer matrix due to the dopant effect, as previously reported.^20,28^ The doped films show three major mass-loss events: the first occurs in the 115 to 160 °C interval for both films, with mass losses of around 16% for the Tb^3+^ film and about 10% for the Eu^3+^ film, which is slightly more thermally stable. The second mass-loss event in the films occurs at an onset temperature of ∼270 °C and reaches its final, most prominent mass-loss at ∼355 °C, with residues corresponding to about 2% mass. In contrast, the undoped film shows only one major thermal event, starting at ∼350 °C and continuing until almost complete degradation.

The FTIR absorption spectra of the [RE_2_(nal)_6_(H_2_O)_3_]·9H_2_O (RE: Eu^3+,^ Gd^3+,^ and Tb^3+^) complexes, sodium nalidixate salt (Na­(nal)·xH_2_O), free ligand (*Hnal*) and the PMMA:(1%)­[RE_2_(nal)_6_] films, were recorded from 4000 to 400 cm^–1^ at room temperature (300 K). In Figure S2a, the vibrational spectra of the RE^3+^-complexes show a broad absorption band with a maximum at 3400 cm^–1^ assigned to the stretching of the O–H bonds of the H_2_O molecules present in all complexes, and the Na­(nal)·xH_2_O salt. On the other hand, the absorption band is absent for the free ligand, indicating that it is completely anhydrous. For the complex spectra, there is a shift in the position of the absorption band corresponding to the stretching of the carbonyl group of the ligand pyridone ring, which shows stretching at 1614 and 1596 cm^–1^ to around 1546 to 1569 cm^–1^ for the ligand corresponding to the sodium salt compared to the coordination compounds, respectively. This spectroscopic data indicate that the carbonyl oxygen atoms participate in coordination to the RE^3+^ ions.^19^ The difference between the stretching of the asymmetric and symmetric stretching bands of the COO^–^ bonds of the carboxylate groups [Δν = ν_as_(COO^–^) – ν_s_(COO^–^)], using the ionic stretching values of the sodium nalidixate salt as a reference for comparison with the complexes, is a valuable tool for determining the coordination modes of carboxylate ligands.^34^

In summary, the Na­(nal)­xH_2_O salt showed a Δν value equal to 278 cm^–1^. In contrast, the complexes all showed Δν ∼ 270 cm^–1^, very close to that of the ionic salt, leading to the conclusion that the coordination mode is a bidentate bridging type, as evidenced by the shifts in both stretching frequencies in the same direction.^34^ It is noteworthy that the coordination modes of the nalidixate ligand showed good agreement with SC-XRD data. In addition, the analysis of the FTIR absorption spectra (Figure S2b) of the PMMA:(1%)­[RE_2_(nal)_6_] (RE: Eu^3+^ and Tb^3+^) films initially showed the disappearance of the broad band in the range of 3700–3000 cm^–1^, suggesting the anhydrous form of the doped films. Moreover, the disappearance of any O–H band in the FTIR spectra may indicate coordination of the polymeric matrix to the RE^3+^ ions, a phenomenon previously reported in the literature.^20,35^

The powder X-ray diffraction (XPD) patterns of the complexes were recorded over a 3–60° range, with the diffractograms shown in Figure S3. The solids exhibited amorphous character, with broad bands observed for all complexes. Full crystallographic data, data collection parameters, and refinement details are provided in Table S1.

Crystallographic data for this structure have been deposited with the Cambridge Crystallographic Data Centre (CCDC) under deposition number 2541394. The molecular structure of the [Tb_2_(nal)_6_(H_2_O)_3_]·9H_2_O complex is shown in Figure 2 with displacement ellipsoids drawn at the 50% probability level. The compound crystallizes in the monoclinic space group *P*2_1_/*n*, a centrosymmetric space group commonly observed for coordination compounds. The asymmetric unit contains two crystallographically independent terbium­(III) centers (Tb1 and Tb2), indicating that the dinuclear unit does not lie on a special position and that distinct coordination environments surround each metal center.

**Figure 2:**
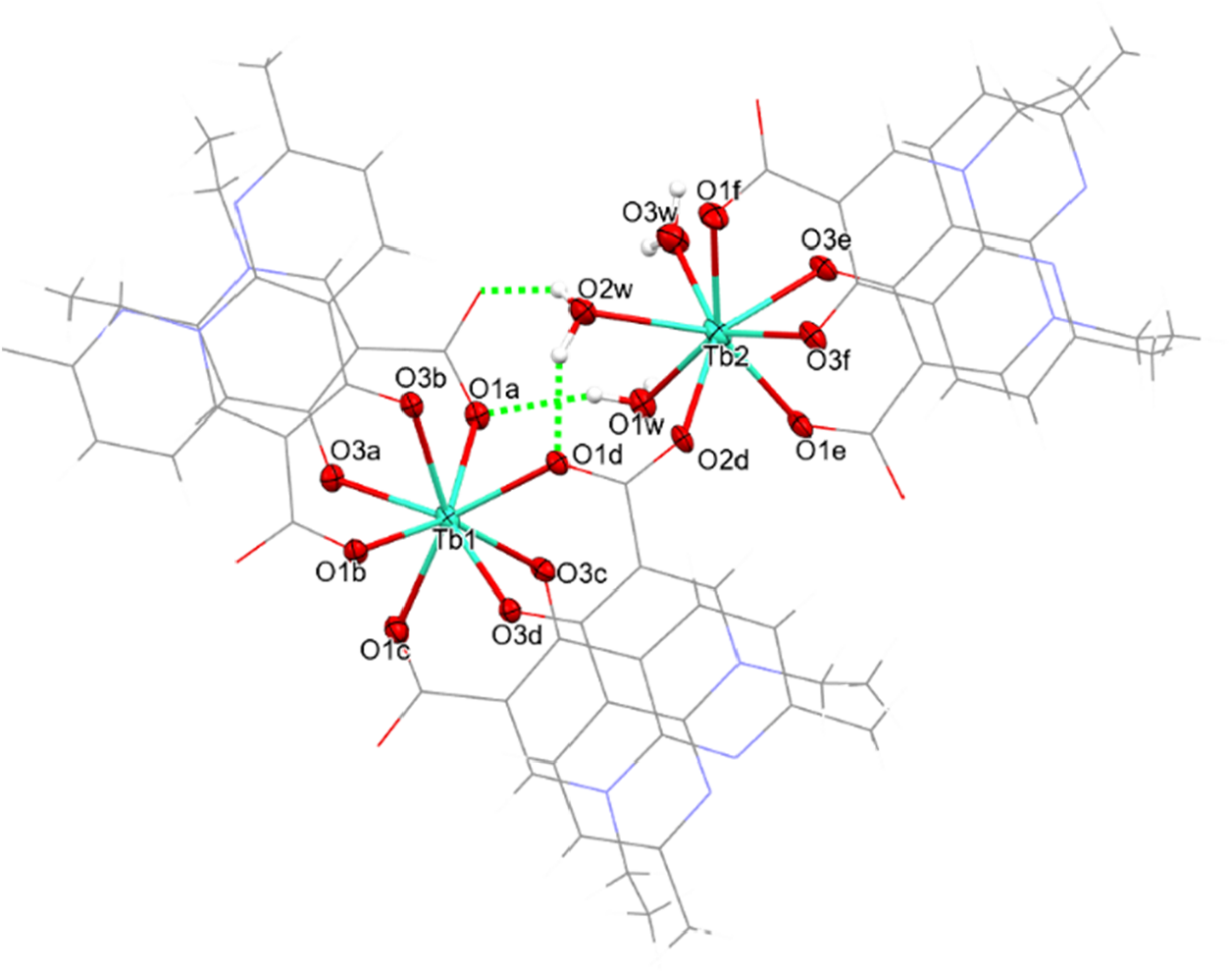
Molecular structure of the dinuclear Tb^3+^ complex with displacement ellipsoids at the 50% probability level. The Tb centers are linked by a μ_2_-oxygen atom (O2d) and a bridging carbonyl group from a nalidixate ligand.

A key structural feature is the presence of a (O2d) from a nalidixate ligand coordinated to Tb1, which acts as a primary bridge toward Tb2, providing the metal–metal connectivity and contributing to the stabilization of the dinuclear core. A detailed inspection of the coordination environments reveals a marked asymmetry between the two metal centers. Four nalidixate ligands coordinate the Tb1 center, each acting in a bidentate fashion through carboxylate and carbonyl oxygen atoms, resulting in a ligand-rich coordination sphere dominated by organic donors. In contrast, the Tb2 center exhibits a more heterogeneous coordination environment, consisting of two nalidixate ligands, three coordinated water molecules, and one additional carbonyl oxygen atom from a nalidixate moiety associated with Tb1. This asymmetric ligand distribution highlights the dual role of the nalidixate ligand, both as a chelating and bridging ligand. The molecular structure is further stabilized by a network of intramolecular hydrogen-bonding interactions involving coordinated water molecules and the oxygen atoms of the nalidixate ligands, contributing to the stabilization of the dinuclear framework. These intramolecular hydrogen bonds play an important role in maintaining the relative orientation of the ligands around the metal centers and in rigidifying the coordination environment, especially at the more solvent-exposed Tb2 center. This effect is consistent with the observed coordination asymmetry between Tb1 and Tb2 and may contribute to the differences in distortion revealed by the SHAPE analysis.

Both terbium centers adopt eight-coordinate geometries defined exclusively by oxygen donor atoms. The coordination geometries were analyzed using the SHAPE program based on continuous shape measures (CShM).^36^ For Tb1, the lowest CShM value corresponds to a biaugmented trigonal prism (JBTPR-8, CShM = 13.01). At the same time, for Tb2, the closest geometry is also a biaugmented trigonal prism (CShM = 11.67), with contributions from triangular dodecahedron and snub diphenoid geometries. These values indicate significantly distorted coordination environments, which are consistent with the flexible coordination behavior of RE^3+^ ions.^37^ Structural disorder is observed in portions of the organic framework and solvent regions, which were modeled using split positions and appropriate geometric restraints. Several oxygen atoms associated with solvent molecules exhibit relatively large displacement parameters, indicating significant positional disorder.

Both symmetry operations govern the crystal packing and an extensive hydrogen-bonding network involving coordinated and lattice water molecules, leading to a three-dimensional supramolecular architecture. The structure contains approximately 25 lattice water molecules per asymmetric unit, which occupy solvent-accessible channels throughout the crystal lattice. Due to their severe disorder and diffuse electron density, these solvent molecules could not be reliably modeled and were therefore omitted from the refinement (see Section 2).

The discrepancy in hydration degree and the loss of crystallinity in bulk powder compared to the single-crystal structure are possibly attributed to thermal dehydration promoted during the drying stage. In the [Tb_2_(nal)_6_(H_2_O)_3_]·25H_2_O system, single-crystal analysis reveals that the 25 lattice water molecules occupy structural channels (Figure S4) and are essential for 3D integrity. Even at relatively low temperatures, the removal of a significant fraction of these molecules triggers a concerted structural collapse. As demonstrated by Takahashi and Uekusa,^38^ the host framework cannot sustain its crystalline packing in the absence of the water-mediated hydrogen-bonding network, leading to the observed amorphization and the reduced hydration (12 H_2_O molecules in total) of the powder sample.

## 4 Results and Discussion

### 4.1 Diffuse Reflectance Spectroscopy (DRS)

The DRS study of the [RE_2_(nal)_6_(H_2_O)_3_]·9H_2_O (RE: Eu^3+^ and Gd^3+^) complexes were recorded at room temperature, using BaSO_4_ as a standard, in the range of 300 to 600 nm, which the complexes show the broad bands assigned to the electronic S_0_ → S_1_ transition of the *nal* ligand as well as narrow bands attributed to the infraconfigurational 4f transitions of the Eu^3+^ ion (^7^F_0_ → ^5^L_6_, ^7^F_0_ → ^5^D_2_ and ^7^F_0_ → ^5^D_1_) (Figure 3a). It is noteworthy that the DRS spectra of the Eu^3+^-complex (Figure 3a red line) are shifted to lower energy compared to those of the Gd^3+^-complex (Figure 3a violet line), indicating the presence of a low-lying ligand-to-metal charge-transfer (LMCT) band.^12,39–41^ The pale yellowish color of the Eu^3+^-complex (Figure 3b) compared with the white color of the Gd^3+^ complex confirms the presence of a LMCT band in the europium complex. Besides, the investigation of the DRS spectra (Figure 3a) revealed the presence of an LMCT band at 26,240 cm^–1^, and was further used to estimate the S_1_ energy level of the ligand (Figure S5). This value was determined from the barycenter of the reflectance band of the Gd^3+^-based complex, yielding an energy of approximately 30,800 cm^–1^, both values were determined using the *JOYSpectra Web Platform* (http://www.joyspectra.com.br)^42^ and the energy transfer process, which will be further elucidated later in Section 4.5. Also, the DRS analysis was applied to the PMMA:(1%)­[RE_2_(nal)_6_] films (RE: Eu^3+^ and Tb^3+^) and an undoped PMMA sample to investigate how dopant incorporation can influence the spectral profiles of the systems. As shown in Figure 3c, the normalized spectra indicate that doping the complexes at 1% (w/w) did not lead to noticeable changes in the profiles, as the areas practically overlapped. However, only minor changes are observed in the UV region, likely due to the low doping concentration of 1%.

**Figure 3:**
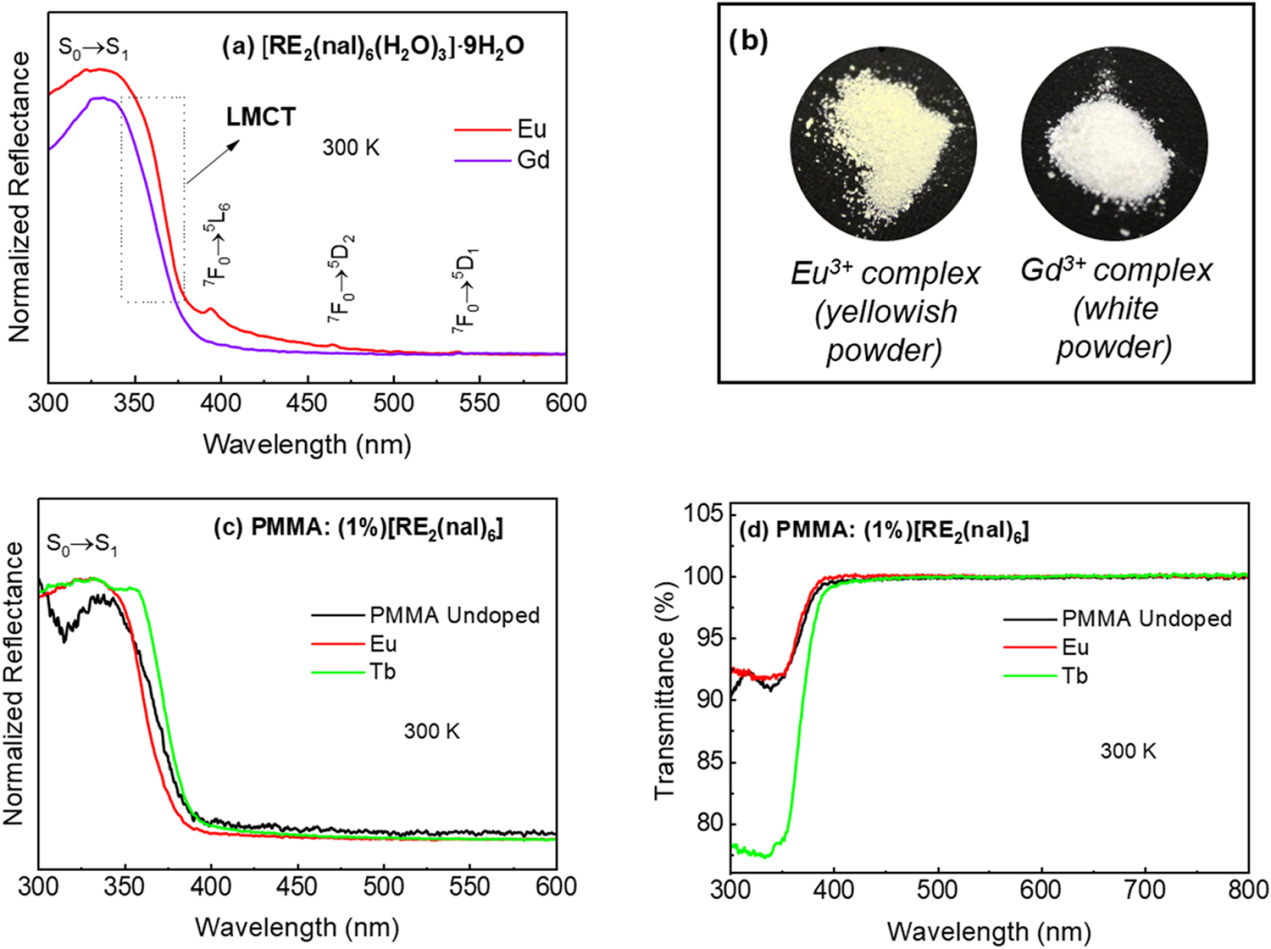
(a) Diffuse reflectance spectra of the [RE_2_(nal)_6_(H_2_O)_3_].9H_2_O complexes, where RE: Eu^3+^ (red line) and Gd^3+^ (purple line) were registered at 300 K. (b) Photograph of complex powders of the Eu^3+^ (yellowish powder) and Gd^3+^ (whitish powder) under ambient light, courtesy of Cezar Guizzo. Copyright 2026. (c) Diffuse reflectance spectra of the undoped PMMA film (black line) and doped films (1%)­[RE_2_(nal)_6_] where RE: Eu^3+^ (red line) and Tb^3+^ (green line) were registered at 300 K. (d) Transmittance spectra of the undoped PMMA film (black line) and doped films (1%)­[RE_2_(nal)_6_] where RE: Eu^3+^ (red line) and Tb^3+^ (green line) were registered at 300 K.

The potential application of polymeric films depends on several factors, including high transmittance in the visible (400–700 nm) and near-infrared (780−2500 nm) regions.^43^ Hence, the transmittance spectral data of the PMMA:(1%)­[RE_2_(nal)_6_] films (RE: Eu^3+^ and Tb^3+^) were recorded in the range of 300 to 800 nm. All doped films exhibit high optical transparency (>95%), confirming that the incorporation of the complexes into the polymeric matrix does not induce significant light scattering and suggesting a homogeneous dispersion of the dopants (Figure 3d). Such optical features, combining high visible-light transmission with efficient UV-to-VIS down-conversion, suggest these films are versatile candidates for photonic coatings and light-management devices, including potential use in LSCs.

### 4.2 Phosphorescent Behavior of Gd^**3+**^ Complex

The energy position of the T_1_ excited state of the *nal* ligand in the [RE_2_(nal)_6_(H_2_O)_3_]·9H_2_O complexes was determined from the time-resolved phosphorescence spectrum of the Gd^3+^-containing compound, due to the strurural energy diagram e paramagnetic effet, recorded at liquid nitrogen temperature (77 K) over the spectral range of 400–750 nm, under excitation at 380 nm (Figure S6). In this case, a 1 ms delay was applied to suppress contributions from the fluorescence band associated with the S_1_ → S_0_ transition. Analogously to the procedure for the DRS spectra, the triplet state (T_1_) energy was estimated as the barycenter using the *JOYSpectra platform* (Figure S7). Therefore, for this case, the T_1_ energy position was determined to be ∼20,008 cm^–1^.

### 4.3 Excitation Investigation of the Eu^**3+**^ and Tb^**3+**^ Systems

The excitation spectra of the [RE_2_(nal)_6_(H_2_O)_3_]·9H_2_O complexes (RE: Eu^3+^ and Tb^3+^) were recorded at 300 K, monitoring the emission at the ^5^D_4_ → ^7^F_5_ (546 nm) and ^5^D_0_ →^7^F_2_ (614 nm) transitions of terbium and europium compounds, respectively (Figure S8). The spectra exhibit similar broad excitation bands in the 300–410 nm range (Figure S8a and b), which originate from intraligand transitions centered on the S_0_ → S_1_ of the *nal* ligand. In addition, the narrow 4f-4f transitions are observed at 370 and 487 nm are assigned to the ^7^F_6_ → ^5^L_10_ and ^7^F_6_ → ^5^D_4_ transitions of the Tb^3+^ ions, respectively (Figure S8a). Furthermore, excitation peaks assigned to the 4f-4f transitions of the Eu^3+^ ion are 393 nm (^7^F_0_ → ^5^L_6_), 464 nm ^7^F_0_ → ^5^D_2_, 525 nm (^7^F_0_ → ^5^D_1_), 534 nm (^7^F_1_ → ^5^D_1_), and 578 nm (^7^F_0_ → ^5^D_0_) have also been observed (Figure S8b).

Analogously, the excitation spectra of the PMMA:(1%)­[RE_2_(nal)_6_] film (Figure S9a and b) were recorded at 300 K for the Eu^3+^ and Tb^3+^ ions. However, the intensities of the excitation broad bands from the overlapped PMMA and *nal* ligand are so high that the intraconfigurational 4f transitions peaks of the Eu^3+^ and Tb^3+^ ions are no longer observable. It is noted that the optical behavior is similar to that reported in the literature for PMMA systems.^12,20^ In general, these excitation data agree with the DRS spectral behavior shown in Figure 3a and 3c.

### 4.4 Luminescence Investigation of the Eu^**3+**^ and Tb^**3+**^ Systems

The emission spectra of the [RE_2_(nal)_6_(H_2_O)_3_]·9H_2_O complexes were recorded at 300 K (Figure 4a and 4c), under excitation in the ligand moiety at 357 and 339 nm for the Eu^3+^ and Tb^3+^ ions, respectively. The phosphorescent broad emission band assigned to the T_1_ → S_0_ (Figure S5) of the *nal* ligands is absent in the emission spectra of the Eu^3+^ and Tb^3+^ complexes (Figure 4a and c), suggesting an efficient *nal* ligand → RE^3+^ ion energy transfer process.

**Figure 4:**
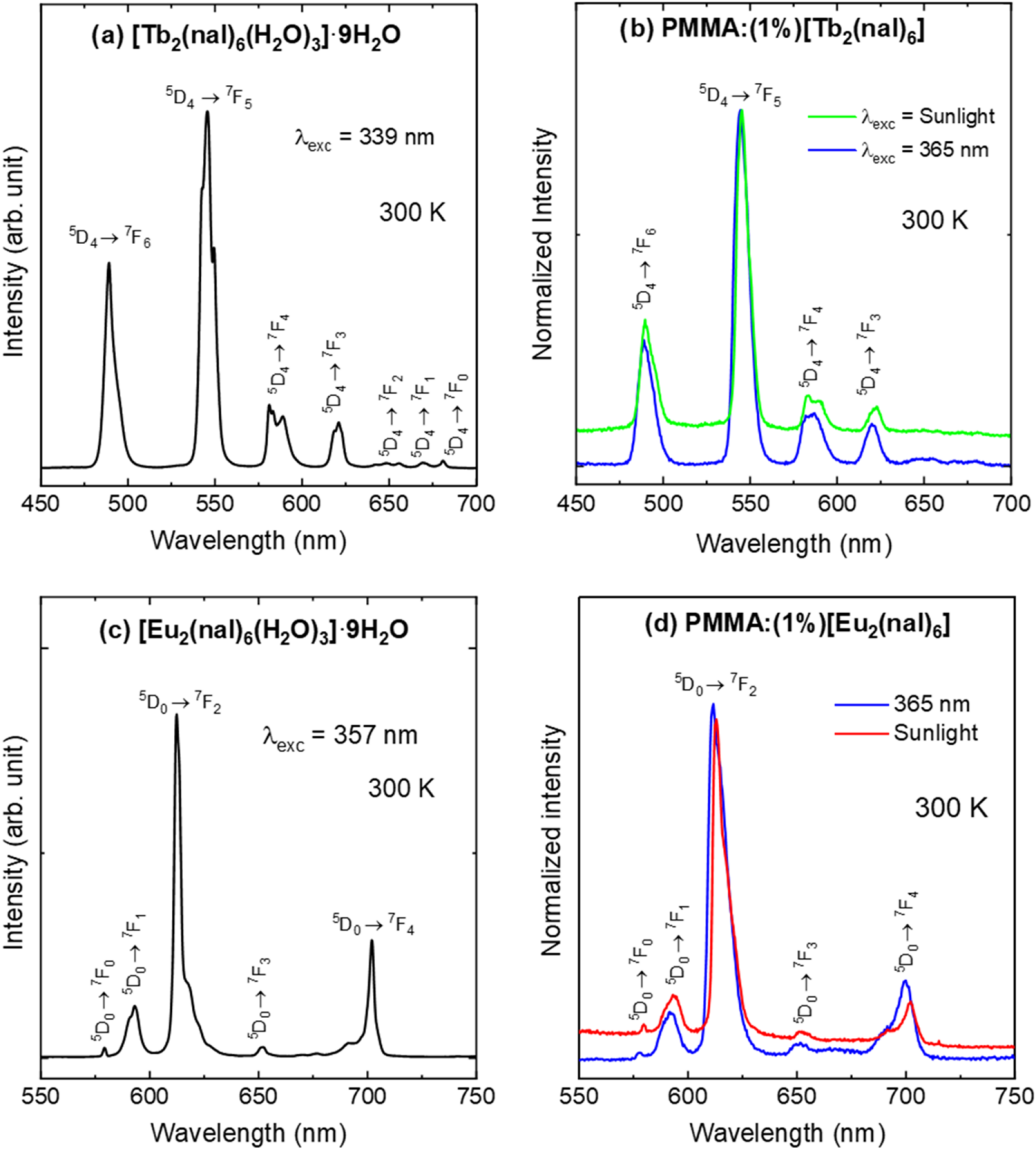
Emission spectra of (a) [Tb_2_(nal)_6_(H_2_O)_3_].9H_2_O complex, excited on 339 nm and (b) PMMA:(1%)­[Tb_2_(nal)_6_] film, under excitation at 365 nm (blue line) and sunlight (green line). Emission spectra of (c) [Eu_2_(nal)_6_(H_2_O)_3_].9H_2_O complex, excited on 357 nm and (d) PMMA:(1%)­[Eu_2_(nal)_6_] film, under excitation at 365 nm (blue line) and sunlight (red line).

The emission spectrum of the Tb^3+^-complex under UVA light excitation at 339 nm (Figure 4a) shows the narrow bands attributed to the ^5^D_4_ → ^7^F_6–0_ transitions characteristic of the Tb^3+^ ion as 489 nm (^5^D_4_ → ^7^F_6_), 546 nm (^5^D_4_ → ^7^F_5_),584 nm (^5^D_4_ → ^7^F_4_), 621 nm (^5^D_4_ → ^7^F_3_), 648 nm (^5^D_4_ → ^7^F_2_), 669 nm (^5^D_4_ → ^7^F_1_) and 681 nm (^5^D_4_ → ^7^F_0_). It is noteworthy that the high emission intensity band is assigned to the ^5^D_4_ → ^7^F_5_ transition.

For the emission spectra of the PMMA:(1%)­[Tb_2_(nal)_6_] film were recorded similarly to the complex at 300 K, using UVA light excitation at 365 nm (Figure 4b, blue line), UVB and UVC (Figure S10a and b) as well as under excitation by the sunlight exposure (Figure 4b, green line) in an open external environment. The emission peaks observed in doped polymeric films are very similar in all cases in terms of peak profiles, and the most prominent one was invariably the one corresponding to the ^5^D_4_ → ^7^F_5_ transition. Indeed, in the sunlight-excited spectrum, the slightly low-intensity broad band in the background of the ^5^D_4_ → ^7^F_6–0_ transitions of the Tb^3+^ ion is attributed to the solar spectrum, as also previously observed for other doped polymeric materials.^12,20^ The broadening of the emission bands of the ^5^D_4_ → ^7^F_6–0_ transitions in doped polymer materials (Figure 4b) arises from nonequivalent Tb^3+^ sites within the PMMA polymer matrix, leading to inhomogeneous broadening of the emission peaks compared to the luminescence spectrum of the Tb^3+^-complex in Figure 4.^44^

The luminescence decay curve of the [Tb_2_(nal)_6_(H_2_O)_3_]·9H_2_O complex was also recorded at 300 K (Figure S12a) under indirect excitation in the ligand moiety at 339 nm, while monitoring the emission at 546 nm corresponding to the ^5^D_4_ → ^7^F_5_ transition. Similarly, the lifetime of the PMMA:(1%)­[Tb_2_(nal)_6_] film was measured (Figure S12b) and found to be 1 ms, higher than the value observed for the complex. These optical features arise because the polymeric matrix can also participate in the energy-transfer process upon coordination to a metal ion^35^ and, as already confirmed by the FTIR spectra, there are no H_2_O molecules in the film, which, as is known, are quenchers of luminescence.^45^ These results explain the higher luminescent potential of this doped PMMA film as a luminescence solar concentrator (LSC).

From now on, we will discuss the emission properties of the Eu^3+^-systems based on emission spectra recorded in the 550–750 nm range at 300 K, under ligand excitation at 357 (Figure 4c), 365 nm (d, blue line), and sunlight exposure (d, red line) for the europium complex and doped films, respectively.

These luminescence spectra of the [Eu_2_(nal)_6_(H_2_O)_3_]·9H_2_O complex exhibit only the sharp emission peaks assigned to the ^5^D_0_ → ^7^F_J_ transitions at 579 nm (^5^D_0_ → ^7^F_0_), 592 nm (^5^D_0_ → ^7^F_1_), 614 nm (^5^D_0_ → ^7^F_2_), 652 nm (^5^D_0_ → ^7^F_3_) and 698 nm (^5^D_0_ → ^7^F_4_) characteristic of the metal ions as well as the absence of the T_1_ → S_0_ *nal* ligand, indicating an efficient *nal* → Eu^3+^ energy transfer process. The hypersensitive ^5^D_0_ → ^7^F_2_ transition is the most intense, and is mainly responsible for the intense red emission arising from the complex, suggesting an increase in the distortion of the chemical environment around the Eu^3+^ ion. It is noteworthy that the (^5^D_0_ → ^7^F_4_) transition of Eu^3+^ ion also shows a high intensity compared to the ^5^D_0_ → ^7^F_1_, suggesting a special case due to the symmetry site which belongs to the D_4d_ point group as reported in Ferreira and co-workers.^46^

The emission spectra of the PMMA:(1%)­[Eu_2_(nal)_6_] film recorded under excitation at 365 nm (UVA) (Figure 4d blue line), 310 nm (UVB) (Figure S11a) and 254 nm (UVC) (Figure S11b) as well as under sunlight exposure (4d red line). For these systems, broader bands of the ^5^D_0_ → ^7^F_J_ transitions are observed when compared to those of the isolated europium complexes (Figure 4c), due to the presence of Eu^3+^ multisites into the polymer matrix, yielding inhomogeneous broadening of the emission peaks. Figure 4d shows that changes in the relative emission intensities between the ^5^D_0_ → ^7^F_J_ and ^5^D_0_ → ^7^F_4_ transitions indicate chemical interactions between the PMMA polymer and the Eu^3+^ complex.

The luminescence decay curves of the [Eu_2_(nal)_6_(H_2_O)_3_]·9H_2_O complex were also recorded at 300 K (Figure S13a and b) under excitation at ^7^F_0_ → ^5^D_2_ transition of the Eu^3+^ ion at 464 while monitoring the emission at 614 nm corresponding to the hypersensitive ^5^D_0_ → ^7^F_2_ transition. In this compound, a lifetime value of 0.58 ms is observed. Also, the lifetime of the PMMA:(1%)­[Eu_2_(nal)_6_] film was measured, exhibiting a decay time of 0.96 ms.

The values of the experimental intensity parameters (Ω_2_ and Ω_4_) for the complex [Eu_2_(nal)_6_(H_2_O)_3_]·9­(H_2_O) are calculated using eq 1

1 $$\Omega_{\lambda }=\frac{4e^{2}\omega^{3}A_{0\to J}}{3{\mathrm{\hbar c}}^{3}\chi \langle^{7}F_{\lambda }|\left|U^{\left(\lambda \right)}\right|{|}^{5}D_{0}\rangle^{2}}$$

In this equation, *e* is the elementary charge of the electron, ω is the transition angular frequency, χ is the Lorentz local field correction, *ℏ* is the reduced Planck constant, and *c* is the speed of light. The values of the squared reduced matrix elements, ⟨^7^F_J_|| U­(λ) ||^5^D_0_⟩^2^, are 0.0032 and 0.0023 for λ = 2 and 4, respectively.^47,48^ The value of χ was calculated based on the medium refractive index as 1.5.^49^ Recent works reveal that the Ω_2_ parameter is sensitive to the symmetry around the Eu^3+^ ion. At the same time, Ω_4_ is more sensitive to the bond distance between the ion and the ligands, i.e., the covalency of the Eu^3+^–O bonds.^50,51^

Furthermore, the values of the Einstein spontaneous emission coefficients (A_0→J_) for the ^5^D_0_ → ^7^F_J_ (J = 0 → 4) transitions were determined from the electronic transitions of the emission of the Eu^3+^ complex, using eq 2

2 $$A_{0\to J}=\left(\frac{S_{0\to J}}{S_{0\to 1}}\right)A_{0\to 1}$$

S_0→J_ corresponds to the areas under the emission curves for the ^5^D_0_ → ^7^F_J_ (J = 2 and 4) forced dipole transitions, and S_0→1_ corresponds to the area under the curve of the ^5^D_0_ → ^7^F_1_ transition, which is magnetic dipole (MD) allowed, with its intensity being practically insensitive to the chemical environment, thus being adopted as a reference.^45^

The value of the intrinsic emission quantum yield (**Q**_**Eu** ^3+^_^**Eu** ^3+^^) is defined as the ratio of radiative decays (*A*_rad_) to total decays (*A*_total_) (eq 3), which corresponds to the sum of radiative and nonradiative decays.^45,49^ Moreover, the *A*_total_ value is obtained from the emission lifetime (τ_obs_), via eq 4, by directly exciting the Eu^3+^ ion at the ^7^F_0_ → ^5^D_2_ transition at 464 nm from the spectra recorded at 300 K.

3 $$Q_{{\mathrm{Eu}}^{3+}}^{{\mathrm{Eu}}^{3+}}=\frac{A_{\mathrm{rad}}}{A_{\mathrm{rad}}+A_{\mathrm{nrad}}}=\frac{A_{\mathrm{rad}}}{A_{\mathrm{total}}}$$

4 $$A_{\mathrm{total}}=A_{\mathrm{rad}}+A_{\mathrm{nrad}}=\frac{1}{\tau }$$

Table 1 contains the obtained values for the complex [Eu_2_(nal)_6_(H_2_O)_3_]·9H_2_O, regarding the intensity parameters Ω_2_ and Ω_4_, *A*_rad_, *A*_nrad_, *A*_total_, τ_obs_, and *Q*_Eu^3+^_^Eu^3+^^. The Ω_2_ parameter of the complex is smaller than in the PMMA:(1%)­[Eu_2_(nal)_6_] film, indicating local distortions of the chemical environment around the Eu^3+^ ion in the polymer system (Table 1), leading to a high intensity of the ^5^D_0_ → ^7^F_2_ transition.^15,52^ In addition, the Ω_4_ parameter of the europium complex is smaller than that of the polymer film, yielding a high structural distortion in the doped film that is dominant.^12,15,22^ As discussed in reference,^46^ this is possibly due to structural features from the Eu^3+^ ion in the D_4d_ symmetry site.

**Table 1:** Experimental Intensity Parameters (Ω_2,4_), Radiative (*A*_rad_), and Non-Radiative (*A*_nrad_) Decay Rates, Emission Lifetime (τ_464_), and Intrinsic Emission Quantum Yield (*Q*_Eu^3+^_^Eu^3+^^) of Eu^3+^ Systems, Parameters Recorded at 300 K

Eu^3+^-system	Ω_2_ (10^–20^ cm^2^)	Ω_4_ (10^–20^ cm^2^)	***A***_**rad**_ (s^–1^)	***A***_**nrad**_ (s^–1^)	***A***_**total**_ (s^–1^)	**τ**_464_ (ms)	***Q***_**Eu** ^3+^_^**Eu** ^3+^^ (%)
[Eu_2_(nal)_6_(H_2_O)_3_]·9H_2_O	8.4	6.1	417	1307	1724	0.580	24
PMMA:(1%)[Eu_2_(nal)_6_]	12.9	7.8	566	480	1046	0.980	54

Despite the high number of water molecules in the [Eu_2_(nal)_6_(H_2_O)_3_].9H_2_O complex, the **Q**_**Eu** ^3+^_^**Eu** ^3+^^ value is consistent with that reported even with β-diketonate ligands from previous works,^54,55^ showing the pivotal role of the intricate hydrogen bonds involving H_2_O molecules, which lead to an exceptional rigidity of the chemical structure, as already discussed above in the single-crystal analysis. The **Q**_**Eu** ^3+^_^**Eu** ^3+^^ values for the PMMA:(1%)­[Eu_2_(nal)_6_] film is higher than in the Eu^3+^ complex, due to the chemical interaction between the polymer and the complex, something already reported in the literature,^28,35,56^ who observed a drastic increase in luminescence efficiency when comparing the precursor Eu^3+^-based complex and the polymeric system, a behavior also in Tb^3+^-doped films.^12,20,57^

### 4.5 Analysis of the Ligand-to-Metal Intramolecular Energy Transfer

Using spectroscopic data of the RE^3+^ ions, it is possible to construct a partial energy-level diagram that indicates the energy transfer from the *nal* ligand to the Eu^3+^ and Tb^3+^ ions. The incident radiation on the ligand excites it from the ground singlet state (S_0_) to the excited singlet state (S_1_), and this energy decays nonradiatively via intersystem crossing (ISC) to the excited triplet state (T_1_) (Figure 5a). The T_1_ state energy of the *nal* ligand is close to but below the ^5^D_4_ emitting level of the Tb^3+^ ion. This small energy gap favors a thermally assisted back-transfer (BT) process from the metal center to the ligand T_1_ state, thereby decreasing the sensitization efficiency via the T_1_ → Tb^3+^ pathway, in agreement with the Latva rule.^58^ On the other hand, the Tb^3+^ complex exhibits intense luminescence, indicating the presence of an alternative and efficient population pathway. In this regard, it is reasonable to assume that energy transfer (ET) occurs from the ligand S_1_ state, located at ∼30,000 cm^–1^, suggesting an efficient ligand-to-metal energy transfer via the exceptional S_1_ → Tb^3+^ process. This optical feature is the best pathway for sensitizing the high green luminescence of the [Tb_2_(nal)_6_(H_2_O)_3_].9H_2_O complex.

**Figure 5:**
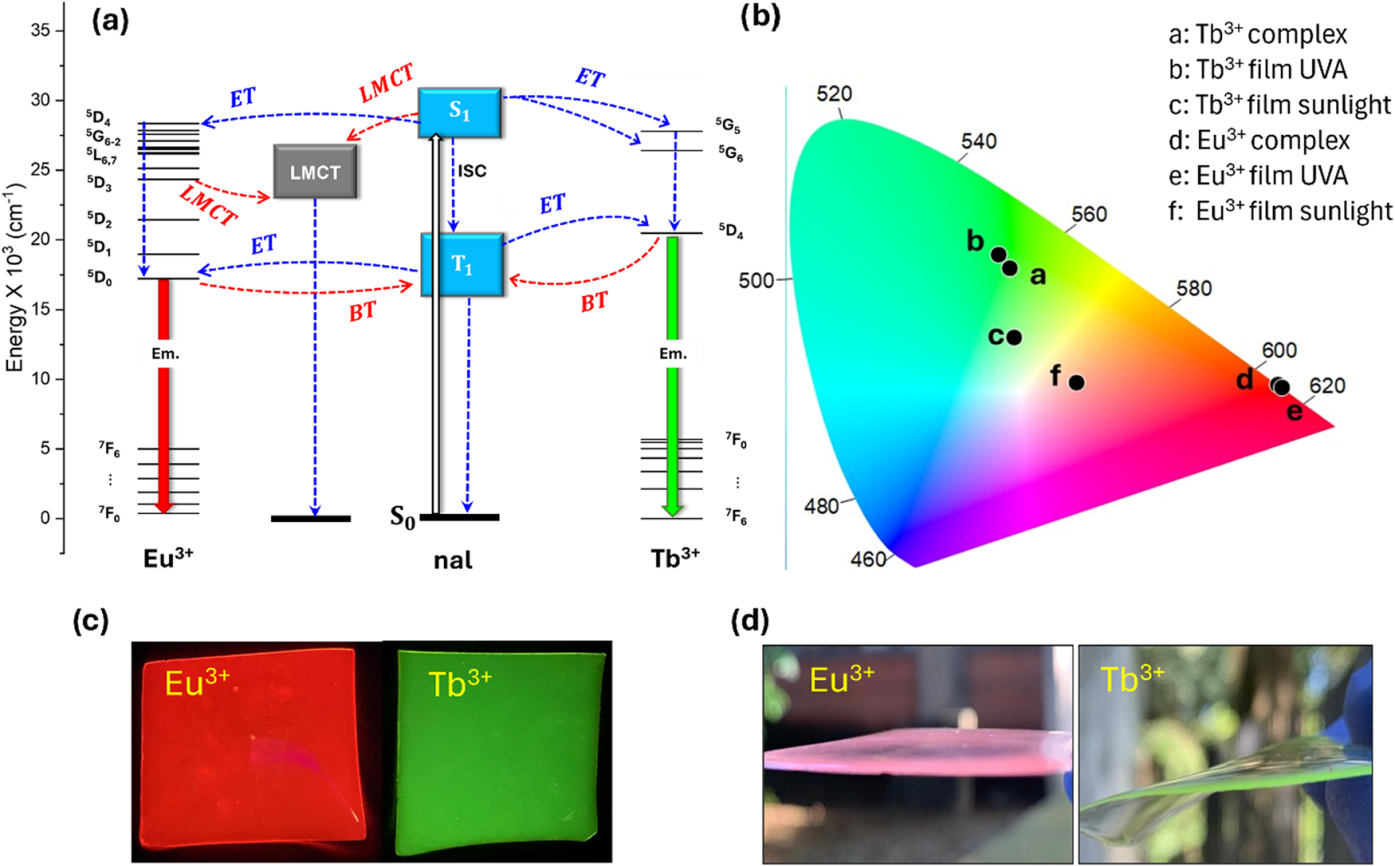
(a) Schematic representation of the energy transfer mechanisms to the Eu^3+^ (left side) and Tb^3+^ (right side) ions. (b) CIE diagram of the [RE_2_(nal)_6_(H_2_O)_3_]·9H_2_O complexes and the (1 wt%) [RE_2_(nal)_6_] doped PMMA films (RE = Eu^3+^ and Tb^3+^); (c) Photographs of the polymeric films under artificial UV light; (d) Photographs of the polymeric films under sunlight exposure.

According to the intramolecular energy transfer process from *nal* ligand to the europium ion in the complex (Figure 5a), it can efficiently sensitize the Eu^3+^ ion via the T_1_ state, which is in resonance. Also, a ligand-to-metal charge-transfer (LMCT) band is observed at ∼26,250 cm^–1^, which lies slightly below the S_1_ state and above the T_1_ state, indicating that the LMCT state can act as a competitive deactivation channel pathway, leading to a quenching contribution of the Eu^3+^ emission described by Faustino and collegues.^59^

Furthermore, the *x*,*y* coordinates of the CIE (*Commission Internationale de l’Eclairage*) chromaticity diagrams (Figure 5b) were determined based on the emission spectra of the complexes [RE_2_(nal)_6_(H_2_O)_3_].9H_2_O, excited in the ligand moiety. Besides, PMMA:(1%)­[RE_2_(nal)_6_] films (RE^3+^: Eu and Tb) were also excited by UVA (365 nm) (Figure 5c) and sunlight (Figure 5d). Regarding the complex and the doped film with the Eu^3+^ ion, the emissions upon excitation in the ligand (UVA) are all located at the red edge of the CIE diagram, indicating the monochromaticity of the complex emissions. The doped film and the complex containing the Tb^3+^ ion, under UV excitation, showed emission coordinates shifted away from the edge in the CIE diagram. Moreover, the luminescence from the Eu^3+^ and Tb^3+^ doped films, when exposed to sunlight, shifts toward the center of the CIE diagram, with a low contribution from background solar radiation,^12^ indicating that these doped polymers can be applied as red and green luminescent solar concentrators.

## 5 Conclusion

The synthesis of the [RE_2_(nal)_6_(H_2_O)_3_]·9H_2_O complexes (RE^3+^: Eu, Gd, and Tb) was successfully performed, according to CHN, TG, and single-crystal XRD data. The data confirmed the formation of RE^3+^ complexes with a 2:6 molar ratio of rare-earth ion to ligand, and FTIR and single-crystal XRD confirmed coordination modes. In PMMA:(1%)­[RE_2_(nal)_6_] films (RE^3+^: Eu and Tb) films, the interaction of the polymer matrix with the complexes was confirmed by FTIR and luminescence spectra. The TG curves indicated reasonable thermal stability of the RE^3+^ complexes, with mass loss of the organic portion at ∼ 300 °C. At the same time, the films showed lower thermal stability due to perturbation of the PMMA stability upon doping these complexes into the polymeric matrix.

The intramolecular energy-transfer process with the Tb^3+^ complex suggests that the S_1_ state of *nal* ligand makes a greater contribution to the sensitization of the metal ion than the T_1_ state, mainly due to the proximity to the ^5^D_4_ emitting levels, leading to a high back-transfer (BT) contribution. In addition, in the Eu^3+^ based complex, the LMCT band contributes slightly to the quenching of the luminescence of the [Eu_2_(nal)_6_(H_2_O)_3_]·9H_2_O complex, through the mild deactivation of the ligand *nal* S_1_ state. Furthermore, although the ligand T_1_ state presents a suitable resonance relative to the ^5^D_0_ level of the Eu^3+^ ion. Nevertheless, the Eu^3+^ complex exhibits a relatively high intrinsic quantum yield of ∼24%, which can be mainly attributed to hydrogen-bonding interactions that rigidify the structure and to the efficient antenna effect.

The PMMA:(1%)­[RE_2_(nal)_6_] films (RE: Eu^3+^ and Tb^3+^) are highly luminescent, which can be ascribed to reduced nonradiative decay pathways arising from the interaction with the PMMA matrix, as well as the absence of coordinated OH oscillators of the water molecules. This effect is further supported by the significant increase in the intrinsic quantum yield (54%) observed for the Eu^3+^ doped in PMMA film relative to the corresponding complex. The polymer films doped with Eu^3+^ and Tb^3+^ ions emit red and green lights, respectively, under sunlight exposure in an open environment. To the best of our knowledge, this photonic feature is unprecedented, making these films strong candidates for use as luminescent solar concentrators (LSCs), as in photovoltaic devices.

## Supplementary Material



## Notes

Thermogravimetric curves of the complexes and PMMA films; infrared absorption spectra of the complexes and PMMA systems; powder X-ray diffraction patterns of the complexes; diffuse reflectance and phosphorescence spectra of the Gd^3+^ complex, in *JOYSpectra web Platform*; excitation, emission, and luminescence decay spectra of the TR^3+^ complexes and PMMA systems; and crystallographic data for [Tb_2_(nal)_6_(H_2_O)_3_]·25H_2_O (CCDC 2541394) (PDF)
