# Supplementary material for: Sunlight-Driven Emission of Eu3+ and Tb3+ Nalidixate Complexes: Achieving Visible Red and Green Light in PMMA Films
Source: ACS Omega. 2026 Jun 30;11(27):40838–49. doi: 10.1021/acsomega.6c04688 (PMC13382690; doi:10.1021/acsomega.6c04688)
Supplement: Supplementary file 1 [file ao6c04688_si_001.pdf]

Support Information

Sunlight-Driven Emission of Eu^{3+} and Tb^{3+} Nalidixate Complexes: Achieving Visible Red and Green Light in PMMA Films

Vitor Waldes[a, b], Paulo R. S. Santos[a], Gabriel A. M. de Oliveira[b], Israel P. Assunção[a, b], João Honorato de Araujo-Neto[a], Iran F. Silva[c], Pedro Miranda Jr[b], Maria Cláudia F. C. Felinto[d], Carlos Lodeiro[e, f], Oscar L. Malta[g], and Hermi F. Brito[a]*.*

[a]Department of Fundamental Chemistry, Institute of Chemistry, University of São Paulo, 05508-900 São Paulo-SP, Brazil.

[b]Education, Science and Technology Federal Institute of São Paulo, 01109-010, São Paulo, Brazil.

[c]Department of Chemistry - University Federal of Paraíba, 58051-970 João Pessoa-PB, Brazil.

[d]Nuclear and Energy Research Institute – IPEN/CNEN, 05508-000 São Paulo-SP, Brazil.

[e]BIOSCOPE Research Group, LAQV - REQUIMTE, Chemistry Department, NOVA School of Science and Technology (FCT NOVA), Universidade NOVA de Lisboa, Caparica 2829-516, Portugal.

[f]PROTEOMASS Scientific Society, 2825-466 Costa de Caparica, Portugal

[g]Department of Fundamental Chemistry, Federal University of Pernambuco, 50670-901 Recife-PE, Brazil.

Corresponding authors: e-mail: hefbrito@iq.usp.br, ipassuncao@ifsp.edu.br.

Summary

Figure S1. **(a)** Thermogravimetric curves of the $[Ln_2(nal)_6(H_2O)_3].9H_2O$ complexes and Hnal ligand, where Ln^{3+}: Eu, Gd and Tb. **(b)** Thermogravimetric curves of the PMMA: (1%)$[Ln_2(nal)_6]$ films, where Ln^{3+}: Eu and Tb. The curves were obtained under a synthetic air of 50 $cm^3 \cdot min^{-1}$ with a constant heating rate of 10 °C min^{-1} from 30 to 900 °C. 3

Figure S2. (a) Infrared absorption spectra of the ligand Hnal, of the salt $Na(nal) \cdot x(H_2O)$, and the complexes $[RE_2(nal)_6(H_2O)_3] \cdot 9(H_2O)$. **(b)** Infrared absorption spectra of the PMMA undoped film and PMMA: (1%)$[RE_2(nal)_6]$, with RE: Eu^{3+} and Tb^{3+}. 3

Figure S3. X-ray powder diffraction (XPD) of the $[RE_2(nal)_6(H_2O)_3].9H_2O$ complexes where RE: Eu^{3+}. 4

Figure S4. Expanded view of the crystal packing of the $[Tb_2(nal)_6(H_2O)_3] \cdot 25H_2O$, highlighting the formation of solvent-accessible channels filled with water molecules. Hydrogen atoms and disordered solvent molecules were omitted for clarity. 4

Figure S5. Diffuse reflectance spectrum of the $[Gd_2(nal)_6(H_2O)_3].9H_2O$ complex. The barycenter of the band was determined (green marker) and used to estimate the ligand S_1 energy level. The shaded area represents the spectral region considered for the barycenter calculation. .5

Figure S6. Phosphorescence spectrum of the complex $[Gd_2(nal)_6(H_2O)_3].9H_2O$. The spectrum was registered at 77 K from 400 to 750 nm. 5

Figure S7. Phosphorescence spectrum of the complex $[Gd_2(nal)_6(H_2O)_3].9H_2O$. he shaded region corresponds to the spectral range considered for analysis. The barycenter of the emission band (green marker) and the full width at half maximum (FWHM) are indicated, providing information on the energetic position and bandwidth of the T_1 state. 6

Figure S8. (a) Excitation spectrum of $[Tb_2(nal)_6(H_2O)_3].9H_2O$ complex and **(b)** Excitation spectrum of $[Eu_2(nal)_6(H_2O)_3].9H_2O$, monitoring the transitions in 546 and 614 nm, respectively. 6

Figure S9. (a) Excitation spectrum of PMMA: (1%)$[Tb_2(nal)_6]$ film and **(b)** Excitation spectrum of PMMA: (1%)$[Eu_2(nal)_6]$ film, monitoring the transitions in 546 and 614 nm, respectively. 7

Figure S10. Emission spectrum of the PMMA: (1%)$[Tb_2(nal)_6]$ films, recorded at 300 K, under excitation at 310 nm **(a)** (UVB radiation) and 254 nm **(b)** (UVC radiation) in the 550−750 nm range. 7

Figure S11. Emission spectrum of the PMMA: (1%)$[Eu_2(nal)_6]$ films, recorded at 300 K, under excitation at 310 nm **(a)** (UVB radiation) and 254 nm **(b)** (UVC radiation) in the 450−700 nm range 8

Figure S12. **(a)** Luminescence decay curves of complex $[Tb_2(nal)_6(H_2O)_3].9H_2O$ recorded at 300 K and **(b)** Luminescence decay curves of PMMA: (1%)$[Tb_2(nal)_6]$ recorded at 300 K. All spectra monitoring the emission of Tb^{3+} with excitation at 339 nm 8

Figure S13. (a) Luminescence decay curves of complex $[Eu_2(nal)_6(H_2O)_3].9H_2O$ recorded at 300 K and **(b)** Luminescence decay curves of PMMA: (1%)$[Eu_2(nal)_6]$ recorded at 300 K. All spectra monitoring the emission of Tb^{3+} with excitation at 464 nm. 9

Table S1. Crystal data and structure refinement. 9

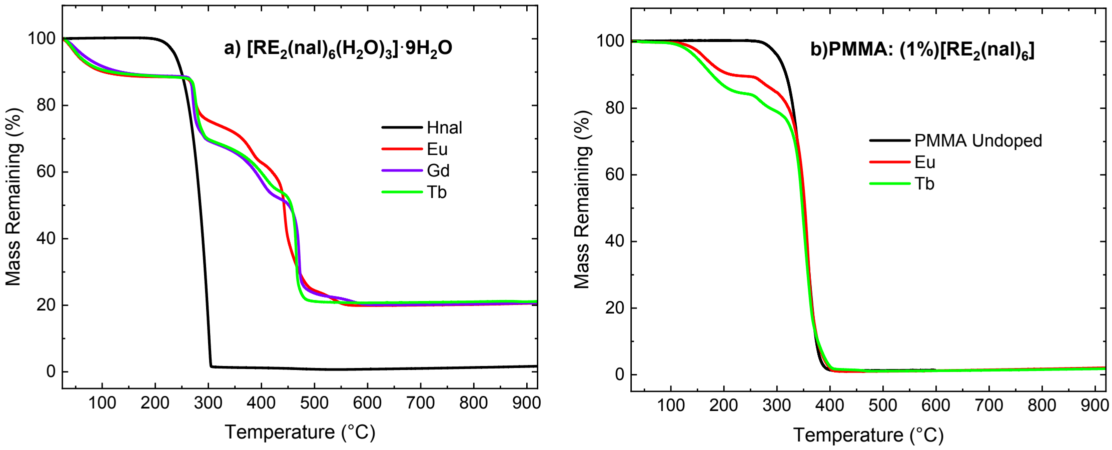

Figure S1. **(a)** Thermogravimetric curves of the $[Ln_2(nal)_6(H_2O)_3].9H_2O$ complexes and Hnal ligand, where Ln^{3+}: Eu, Gd and Tb. **(b)** Thermogravimetric curves of the PMMA: (1%)$[Ln_2(nal)_6]$ films, where Ln^{3+}: Eu and Tb. The curves were obtained under a synthetic air of 50 $cm^3 \cdot min^{-1}$ with a constant heating rate of 10 °C min^{-1} from 30 to 900 °C.

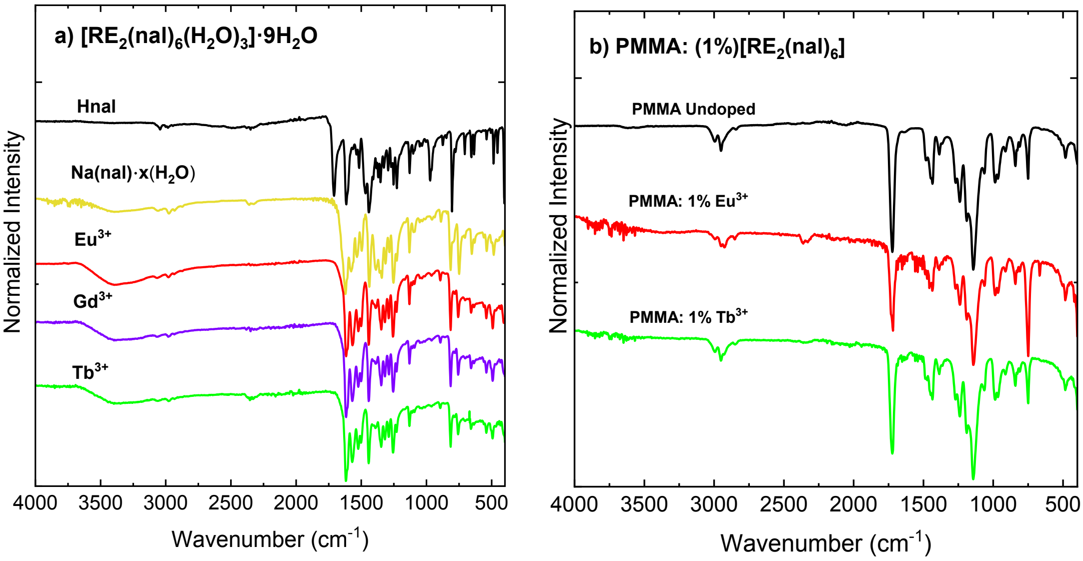

Figure S2. (a) Infrared absorption spectra of the ligand Hnal, of the salt $Na(nal)\cdot x(H_2O)$, and the complexes $[RE_2(nal)_6(H_2O)_3]\cdot 9(H_2O)$. **(b)** Infrared absorption spectra of the PMMA undoped film and PMMA: (1%)$[RE_2(nal)_6]$, with RE: Eu^{3+} and Tb^{3+}.

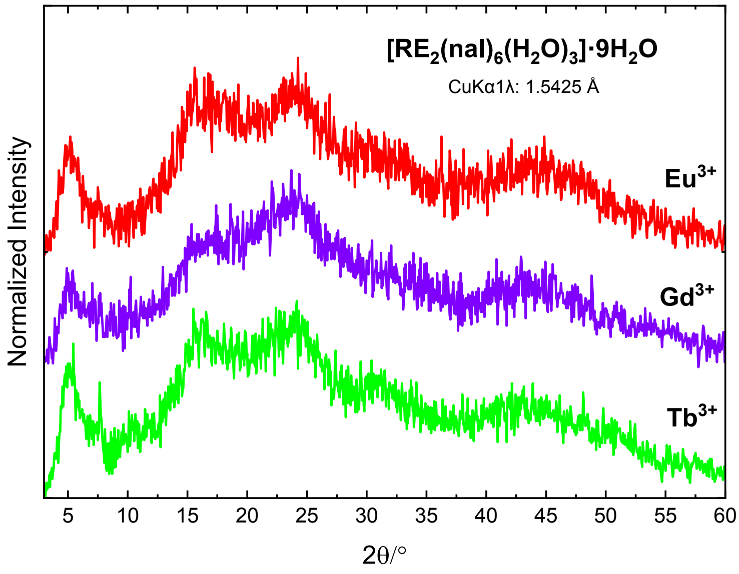

Figure S3. X-ray powder diffraction (XPD) of the $[RE_2(nal)_6(H_2O)_3].9H_2O$ complexes where RE: Eu^{3+} (red line), Gd^{3+} (violet line), and Tb^{3+} (green line), recorded at room temperature in the 3−60° range.

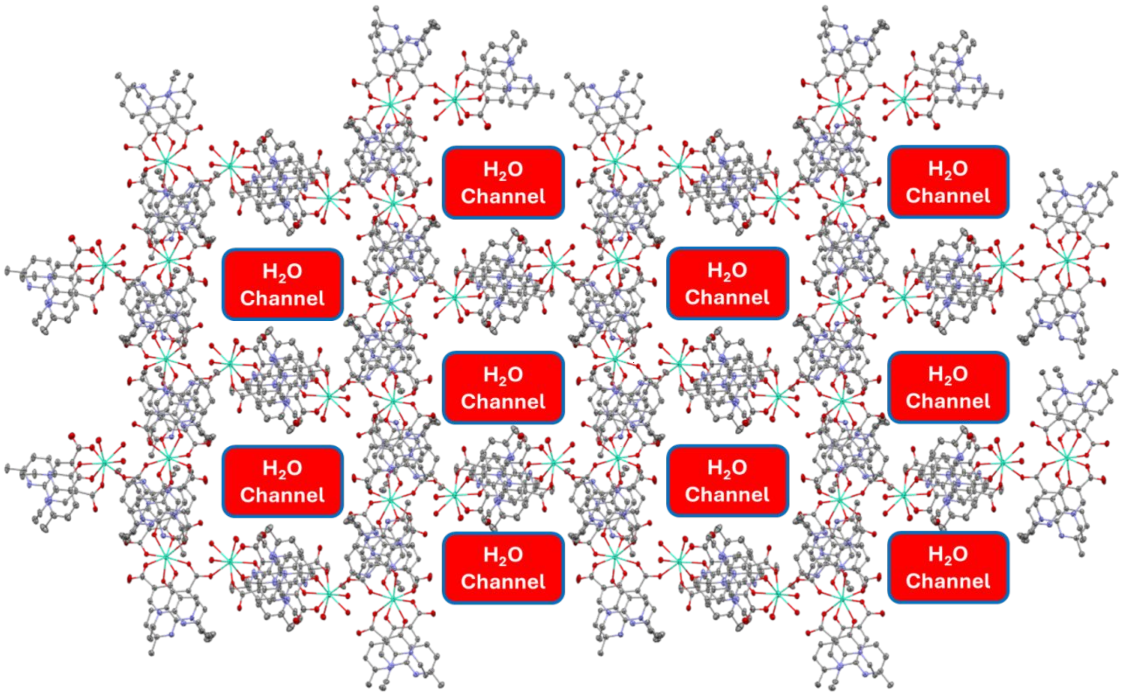

Figure S4. Expanded view of the crystal packing of the $[Tb_2(nal)_6(H_2O)_3]\cdot 25H_2O$, highlighting the formation of solvent-accessible channels filled with water molecules. Hydrogen atoms and disordered solvent molecules were omitted for clarity.

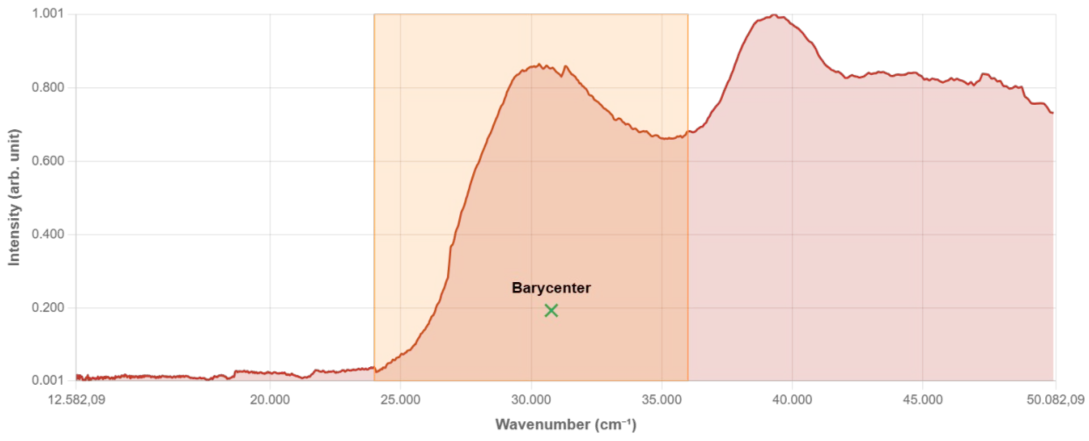

Figure S5. Diffuse reflectance spectrum of the $[Gd_2(nal)_6(H_2O)_3].9H_2O$ complex. The barycenter of the band was determined (green marker) and used to estimate the ligand S_1 energy level. The shaded area represents the spectral region considered for the barycenter calculation.

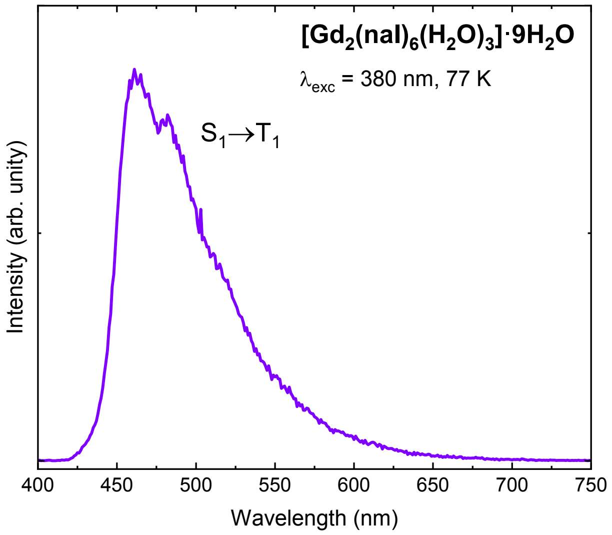

Figure S6. Phosphorescence spectrum of the complex $[Gd_2(nal)_6(H_2O)_3].9H_2O$. The spectrum was registered at 77 K from 400 to 750 nm.

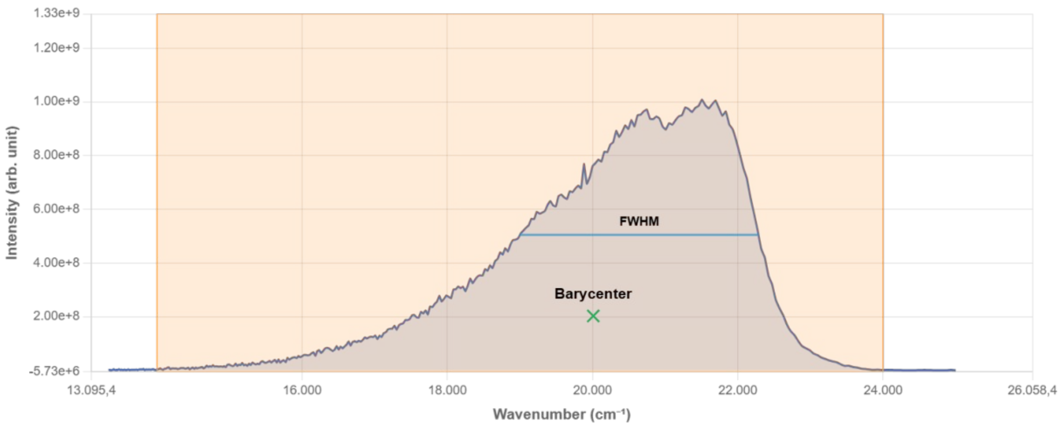

Figure S7. Phosphorescence spectrum of the complex $[Gd_2(nal)_6(H_2O)_3].9H_2O$. he shaded region corresponds to the spectral range considered for analysis. The barycenter of the emission band (green marker) and the full width at half maximum (FWHM) are indicated, providing information on the energetic position and bandwidth of the T_1 state.

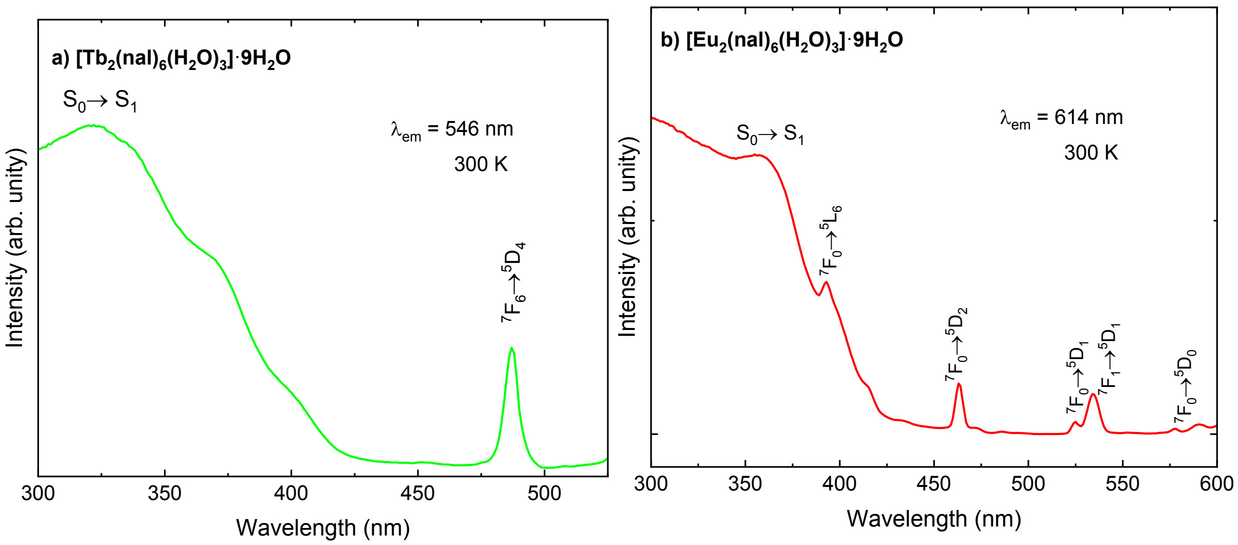

Figure S8. (a) Excitation spectrum of $[Tb_2(nal)_6(H_2O)_3].9H_2O$ complex and **(b)** Excitation spectrum of $[Eu_2(nal)_6(H_2O)_3].9H_2O$, monitoring the transitions in 546 and 614 nm, respectively.

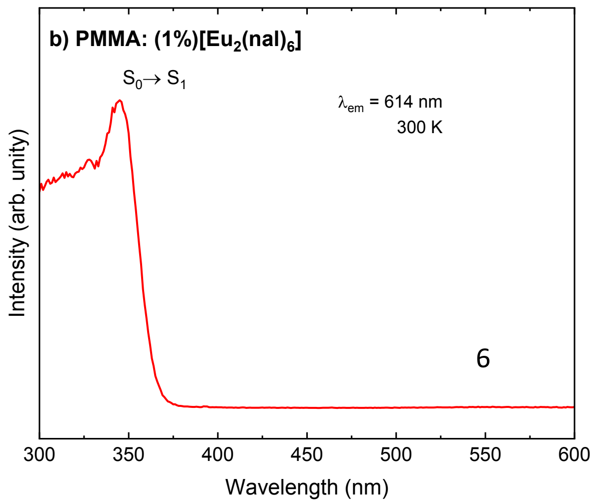

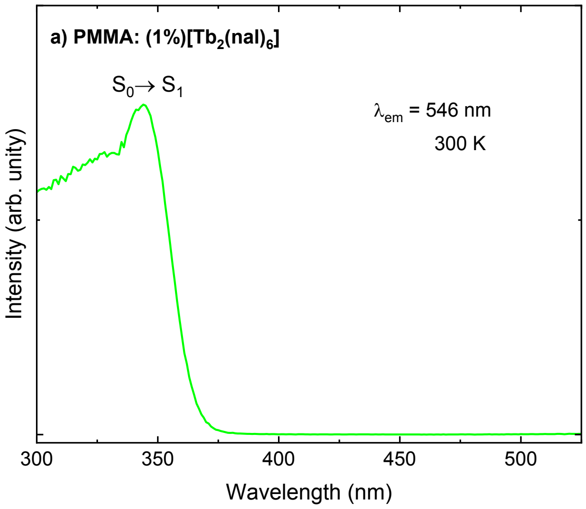

Figure S9. (a) Excitation spectrum of PMMA: (1%)[Tb_2(nal)$_6$] film and **(b)** Excitation spectrum of PMMA: (1%)[Eu_2(nal)$_6$] film, monitoring the transitions in 546 and 614 nm, respectively.

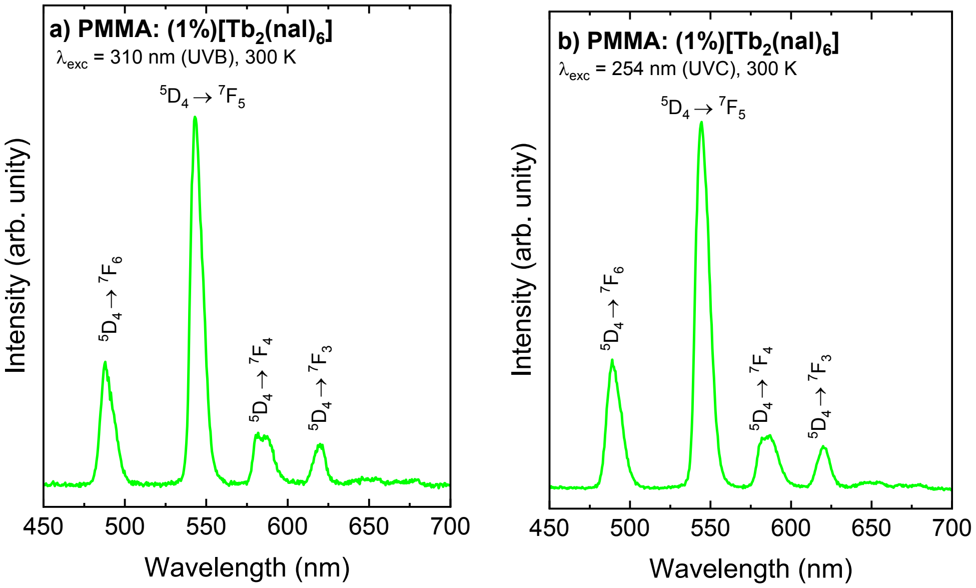

Figure S10. Emission spectrum of the PMMA: (1%)[Tb_2(nal)$_6$] films, recorded at 300 K, under excitation at 310 nm **(a)** (UVB radiation) and 254 nm **(b)** (UVC radiation) in the 550−750 nm range.

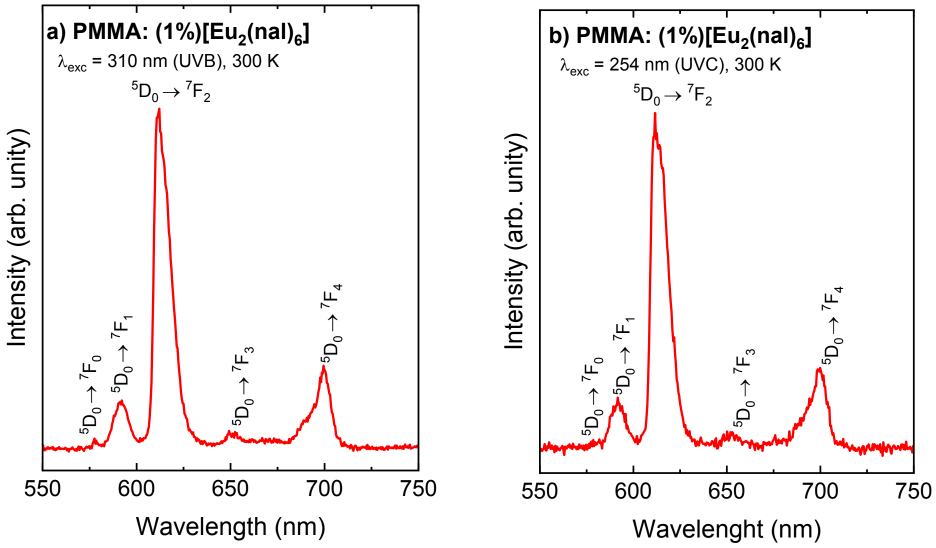

Figure S11. Emission spectrum of the PMMA: (1%)[Eu_2(nal)$_6$] films, recorded at 300 K, under excitation at 310 nm **(a)** (UVB radiation) and 254 nm **(b)** (UVC radiation) in the 450−700 nm range.

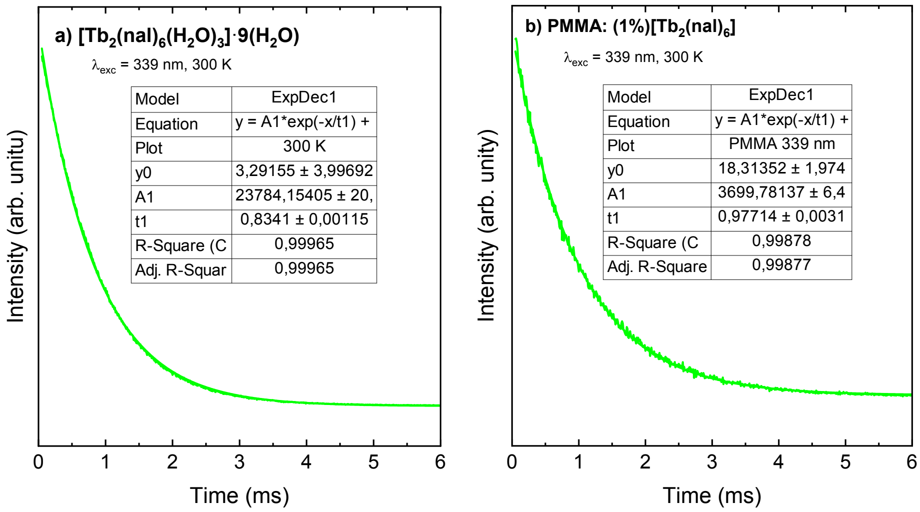

Figure S12. **(a)** Luminescence decay curves of complex [Tb_2(nal)$_6$(H_2O)$_3$].9H_2O recorded at 300 K and **(b)** Luminescence decay curves of PMMA: (1%)[Tb_2(nal)$_6$] recorded at 300 K. All spectra monitoring the emission of Tb^{3+} with excitation at 339 nm.

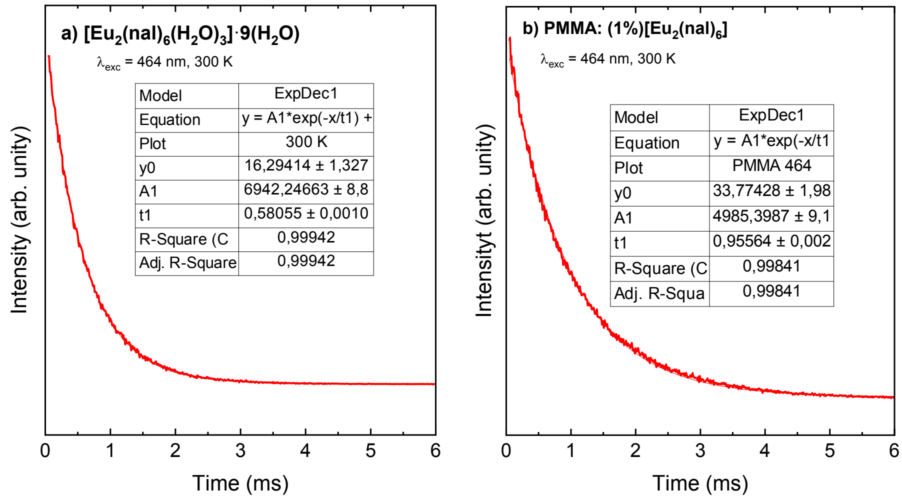

Figure S13. **(a)** Luminescence decay curves of complex $[Eu_2(nal)_6(H_2O)_3].9H_2O$ recorded at 300 K and **(b)** Luminescence decay curves of PMMA: (1%)$[Eu_2(nal)_6]$ recorded at 300 K. All spectra monitoring the emission of Tb^{3+} with excitation at 464 nm.

Table S1. Crystal data and structure refinement.

CCDC code	2541394
Empirical formula	$C_{72}H_{122}N_{12}O_{46}Tb_2$
Formula weight	2209.676
Temperature/K	99.9(4)
Crystal system	monoclinic
Space group	$P2_1/n$
a/Å	14.8223(1)
b/Å	39.7123(3)
c/Å	17.9537(2)
α/°	90
β/°	103.693(1)
γ/°	90
Volume/Å^3	10267.68(16)
Z	4
ρ_{calc}g/cm^3	1.429
μ/mm^{-1}	7.484

F(000)	4493.4
Crystal size/mm^3	0.2 × 0.11 × 0.1
Radiation	Cu Kα (λ = 1.54184)
2Θ range for data collection/°	8.9 to 140.16
Index ranges	$-18 \leq h \leq 15$, $-50 \leq k \leq 50$, $-22 \leq l \leq 22$
Reflections collected	141066
Independent reflections	19464 [R_{int} = 0.0786, R_{sigma} = 0.0446]
Data/restraints/parameters	19464/4/1050
Goodness-of-fit on F^2	1.031
Final R indexes [I>=2σ (I)]	R_1 = 0.0605, wR_2 = 0.1543
Final R indexes [all data]	R_1 = 0.0648, wR_2 = 0.1565
Largest diff. peak/hole / e Å^{-3}	1.70/-1.40
